# EBV-encoded RNA via TLR3 induces inflammation in nasopharyngeal carcinoma

**DOI:** 10.18632/oncotarget.4552

**Published:** 2015-07-04

**Authors:** Zhi Li, Yumei Duan, Shiyue Cheng, Yan Chen, Yanxin Hu, Lu Zhang, Jiang He, Qiong Liao, Lifang Yang, Lun-Quan Sun

**Affiliations:** ^1^ Center for Molecular Medicine, Xiangya Hospital, Central South University, Changsha, China 410008; ^2^ College of Veterinary Medicine, China Agricultural University, Beijing, China 100193; ^3^ Cancer Research Institute, Central South University, Changsha, China 410008

**Keywords:** EBV encoded RNAs (EBERs), TLR3, inflammation, nasopharyngeal carcinoma

## Abstract

Pathogen-induced inflammation has been one of the intensive research areas in carcinogenesis. EBV encoded RNAs (EBERs) have been suggested to play roles in anti-apoptosis and growth-promotion in lymphoid and immune disorders. However, pathological roles of EBERs in solid tumors of epithelia origin remain to be elucidated. Given their characteristic dsRNA structures, recent studies provided evidences for the activation of some pattern recognition receptors (PRR) by EBERs, which is fundamental in the process of pathogenesis. Here, we show that EBERs induce inflammatory response in nasopharyngeal carcinoma (NPC) cells through Toll-like receptor 3 (TLR3), mainly featured by high level of TNFα production. Interestingly, EBERs and EBV latent membrane protein 1 (LMP1) form a positive regulatory loop with NF-κB as a key node that amplifies the inflammatory signals in EBV infected epithelial cells. We demonstrate *in vivo* that EBERs can interact with TLR3 and induce tumor cells to produce cytokines in B16 synergetic tumor and human NPC xenograft models, in which macrophages are recruited and activated, leading to a favorable microenvironment for solid tumor growth. Lastly, we verify a positive association between EBER and TNFα levels in NPC clinical samples and the combination of EBER and TNFα expressions provides a predictor of poor survival of NPC patients. In conclusion, EBERs play a pivotal role in inflammation-to-oncogenesis transition in NPC development.

## INTRODUCTION

Inflammation is a fundamental physiologic process required for wound repair and resolution of infection. However, unresolved or aberrantly regulated inflammation can initiate and propagate carcinogenesis [1]. This process can be either localized within tumor aggregates or present within the stromal microenvironment where tumors are sustained. The most straightforward cause of nonresolving inflammation is the persistence of inflammatory stimuli of exogenous origin, including tumor viruses [2]. The virally infected cells can either be eliminated via cell-mediated apoptosis or persist in a state of chronic infection that leads to oncogenesis. Conservative estimates indicate that at least 20% of all cancer cases are attributable to tumor viruses [3], including Epstein-Barr virus (EBV), Human papillomaviruses (HPV), Hepatitis B virus (HBV) Hepatitis C virus (HCV), Kaposi's sarcoma-associated herpesvirus (KSHV), Merkel cell polyomavirus (MCPV) and Human T-lymphotropic virus 1 (HTLV1), which underscore the importance of defining specific roles of tumor viruses-induced inflammation in initiation and promotion of malignancies.

EBV is a human gamma herpesvirus commonly carried in the majority of the human population and is known to be linked to nearly all cases of nasopharyngeal carcinoma (NPC), along with other malignancies such as gastric cancer, Burkitt's lymphoma, Hodgkin's lymphoma, nasal T-cell lymphoma, and immunoblastic lymphomas in posttransplant and AIDS patients [4]. In addition to various viral proteins involved in viral latency and oncogenesis, EBV encodes two small RNAs (EBER1 and EBER2) that are non-polyadenylated, noncoding and expressed abundantly in all forms of cells latently infected with EBV. EBERs are reported to be involved in several cellular activities such as inhibition of apoptosis [5], prevention of protein translation arrest [6] and may even be involved in transforming normal lymphocytes [7]. Intriguingly, all these functions are associated with EBERs’ unique double-strand RNA secondary structures, which facilitate interaction with dsRNA recognition proteins such as La, PKR, L22, RIG-I and TLR3 [8–12]. However, most of the recognized functions of EBERs mentioned above were identified and studied in B cell lymphoma and the contributions of EBERs to malignancies in epithelial tumors remain poorly understood.

To elucidate the roles of EBERs in NPC, one of the major solid tumors associated with EBV infection, we propose that EBERs are both an inducer of chronic inflammation via TLR3 pathway and a promoter of oncogenesis through interaction with the EBV oncoproteins and cellular modulators. We demonstrate that EBERs can induce expression of pro-inflammatory cytokines in NPC cells, which is dependent on TLR3 activation. We find that the EBER-TLR3 signaling can be amplified through cooperation with the latent membrane protein 1 (LMP1) of EBV. We show that EBERs can trigger an intrinsic inflammatory response to build up a protumorigenic microenvironment as demonstrated in NPC xenograft model and TLR3 knockout mice. Furthermore, we present data from the NPC tissue arrays showing that there is a significant association between EBER expression and TNFα level, and the combined EBER/TNFα expression is a predictor of the patient poor survival. Our results suggest critical roles of EBERs in inflammation-to-oncogenesis transition leading to tumor development of NPC.

## RESULTS

### EBERs trigger TNFα-centered chronic inflammatory response in nasopharyngeal carcinoma cells

**Figure 1:**
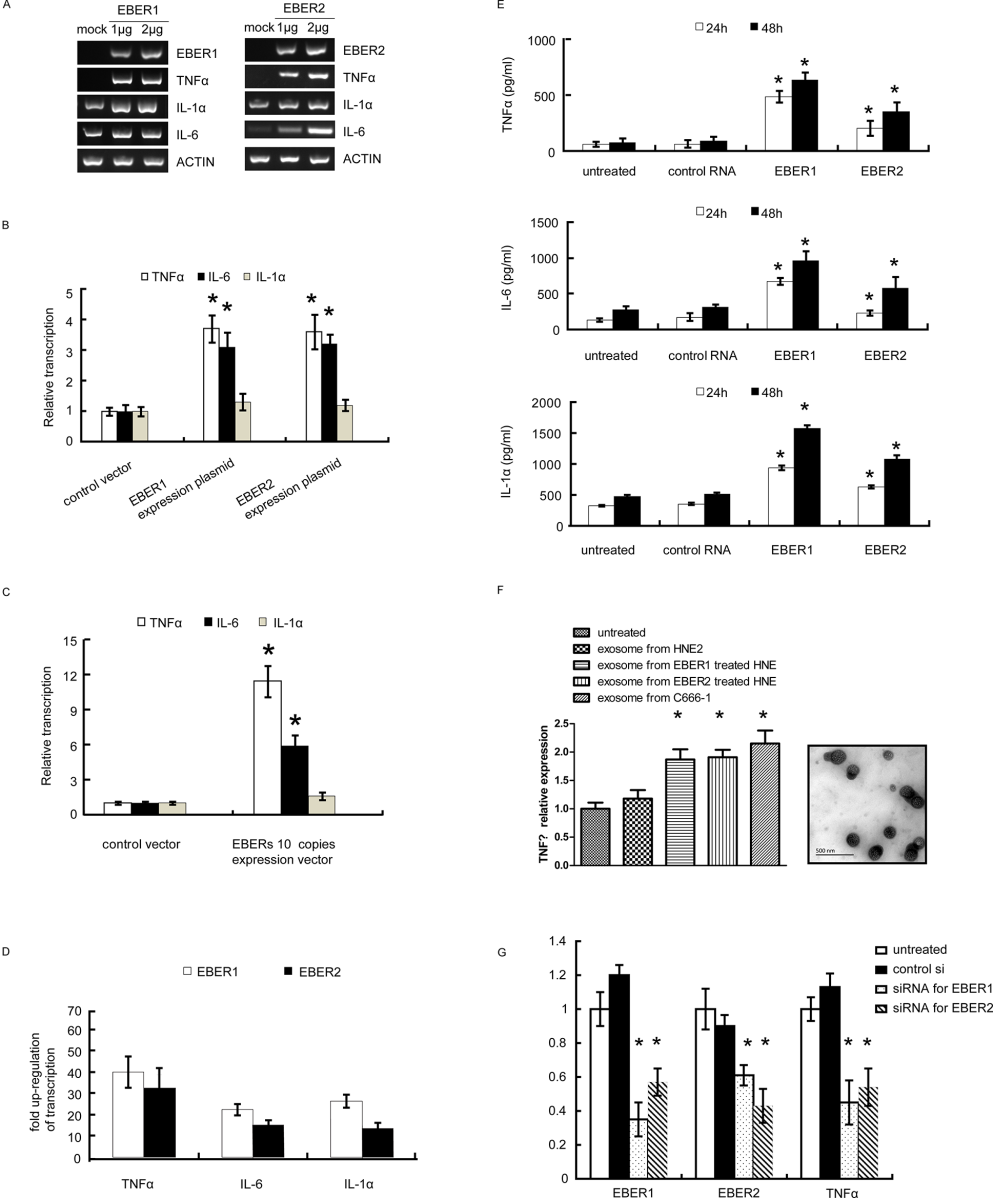
EBERs trigger inflammatory response in NPC cell lines **A.** mRNA levels of inflammatory cytokines (TNFα, IL-1α and IL-6) were determined by RT-PCR in HNE2 cells transfected with different doses of EBER1 (left panel) or EBER2 (right panel) expression plasmids for 24 h. Mock is the vector control. **B.** mRNA levels of inflammatory cytokines were measured by qPCR in HNE2 cells stably expressing single-copy EBER1 or EBER2 maintained in G418 supplemented medium. **C.** HNE2 cells stably expressing 10 tandem copies of EBER1 and EBER2 were subjected to qPCR for mRNA levels of inflammatory cytokines. **D.** Inflammatory cytokine expression triggered by *in vitro* transcribed EBER1 or EBER2. Results were presented as a fold of increase relative to the corresponding EBER plasmids. **E.** HNE2 cells were stimulated with *in vitro* transcribed EBER1 or EBER2. TNFα, IL-6 and IL-1α released into the culture supernatant were quantified by ELISA. **F.** (Left panel), exosomes were isolated from cell culture supernatant of C666–1 or 2 μg *in vitro* transcribed EBERs treated HNE2 cells, and then approximate equal amount of exosomes determined by TEM were applied to challenge untreated HNE2 cells. TNFα transcripts was determined by q-PCR. (Right panel), representative TEM images of exosomes from C666–1 cells culture supernatant. **G.** siRNA targeting EBER1 or EBER2 was transfected into C666–1 cells. mRNA levels of EBER1, EBER2 and TNFα were measured 72 h post transfection. All the data are from 3 independent experiments and representative data are expressed as mean ± SD. In (B), (C), (E), (F) and (G), *P < 0.05 versus control or untreated group.

To investigate the link between the EBER expression and inflammatory responses, we exogenously expressed EBER 1 and EBER2 respectively in an EBV-negative NPC cell line HNE2. As shown in Figure 1A, transient transfection with the EBER expression plasmids into HNE2 cells led to a marked increase of inflammatory cytokine expression including TNFα, IL-6 and IL-1α. To mimic chronic inflammatory response in cells, we stably expressed EBERs in HNE2 cells and found that TNFα and IL-6 were moderately up-regulated, while IL-1α was unaffected (Figure 1B). Previous studies in Burkitt's lymphoma cells suggested that EBERs needed to be maintained at a constant high level to exert their cellular function [13]. Thus, an expression cassette with 10 EBER copies was constructed and introduced into HNE2 to augment the amount of EBER transcripts. To validate the biological relevance of the generated HNE2 cells, we compared the levels of EBERs in C666–1 and HNE2-EBERs-10 copies cells. The EBERs transcripts in HNE2-EBERs-10 copies were found to be approximately 80% relative to the level of EBV-positive cells (C666–1) (Supplementary Figure S1). In this EBER-10-copy-cell line, TNFα was the most upregulated cytokine by EBERs, which increased approximately 10 fold relative to the control cells (Figure 1C).

To rule out the possibility that plasmid rather than EBERs themselves triggered the inflammatory response via different cellular sensors [14–16], *in vitro* transcribed EBER1 or EBER2 were applied to challenge HNE2 cells with *in vitro*-produced tRNA as a control. 0.6 μg or 0.1 μg of *in vitro* transcribed EBER1 or EBER2 (refer to materials and methods) was applied to challenge HNE2 cells seeded in 6 wells. When the EBERs were recovered from the transfected HNE2 cells, the level of EBERs was comparable to that in C666–1 cells (Supplementary Figure S2). As determined by quantitative PCR (qPCR), much more inflammatory cytokines were transcribed (especially TNFα) compared to the cells transfected with the EBER expressing plasmids (Figure 1D). Consistent with this result, enzyme linked immunosorbent assay (ELISA) further showed that IL1α, IL6 and TNFα released into the cell supernatant increased sharply in response to the *in vitro* transcribed EBER1 or EBER2 (Figure 1E).

Recent report demonstrates that EBERs can be released into cell culture supernatant by means of exosomes [17]. Bear this in mind and to further consolidate our observations, we propose that EBER may function via autocrine or paracrine manners by its incorporating into exosomes, which in turn interact with both cancer and non-cancerous cells in tumor microenvironment. To test this, we demonstrated the existence of exosomes in the culture supernatants from HNE2 cells challenged with *in vitro* transcribed EBERs or C666–1 cells. When HNE2 cells were treated with approximately equal amount of exosomes including comparable EBERs (Supplementary Figure S3) from both supernatants, TNF transcription was found to be triggered by the exosomes containing EBERs (Figure 1F).

We next examined the effect of EBERs on the cytokine expression in NPC cells by knockdown of endogenous EBER expression. Targeted knockdown of EBER1 and EBER2 in C666–1 cells was achieved by synthetic siRNAs (Figure 1G). qPCR and ELISA measurements showed that either EBER1 or EBER2 down-regulation led to significant reduction of TNFα transcripts in C666–1 cells (Figure 1G). To our surprise, siRNA targeting EBER1 or EBER2 could reduce both the transcripts of EBER1 and EBER2, which may be due to that EBER1 and EBER2 co-exist as a primary transcript before possible splicing (Supplementary Figure S4). Collectively, these results demonstrate that in NPC cells, either exogenous or endogenous expressions of EBERs induce inflammatory response, mainly featured by high level of TNFα production.

### EBERs induce inflammatory response via TLR3 pathway

**Figure 2:**
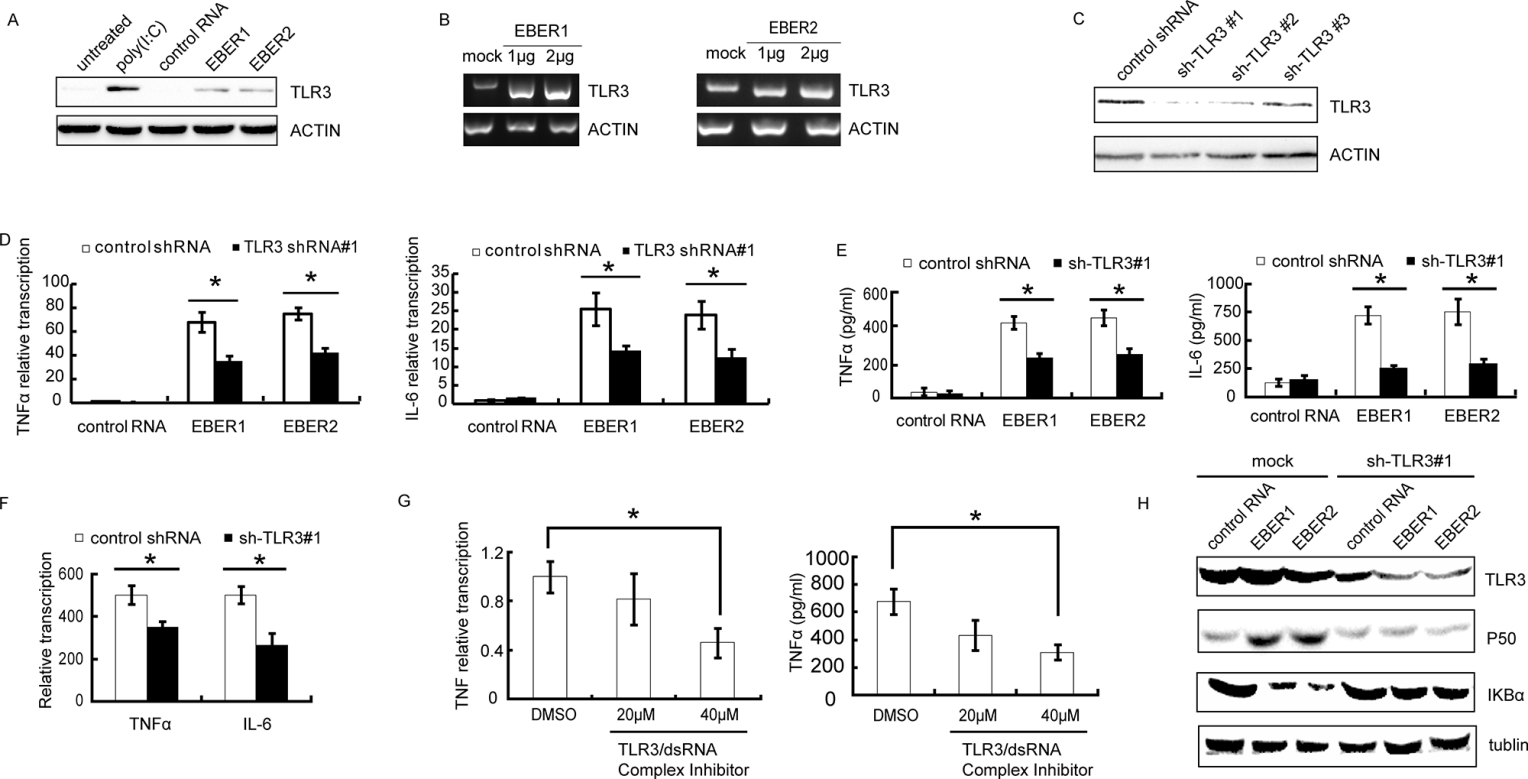
EBERs trigger inflammatory response *in vitro* via TLR3 in NPC cell lines **A.** Protein was extracted and subjected to western blotting to verify TLR3 expression after HNE2 cells were treated with *in vitro* transcribed EBER1 or EBER2. Poly(IC) was used as a positive control and tRNA was used as negative control RNA. **B.** HNE2 cells were challenged with different doses of *in vitro* transcribed EBER1 or EBER2 for 24 h and mRNA level of TLR3 was determined by RT-PCR. **C.** Lenti-virally knockdown TLR3 was verified in HNE2 cells by western blotting. **D.** HNE2 cells transduced with Lenti-shRNA-TLR3 were stimulated with *in vitro* transcribed EBER1 or EBER2. mRNA levels of inflammatory cytokines (TNFα and IL-6) were analyzed by qPCR; TNFα and IL-6 released into the supernatant of cultured HNE2 cells were measured by ELISA as in **E. F.** mRNA levels of TNFα and IL-6 in C666–1 cells transduced with sh-TLR3#1 cells were determined by qPCR 48 h post transduction. **G.** C666–1 cells were challenged with the TLR3-dsRNA complex inhibitor for 48 h and TNFα transcripts and release into the culture supernatant were measured by qPCR (left) or ELISA (right). **H.** p50 and IκBα expression status was verified by western blotting in TLR3 knockdown or mock treated HNE2 cells after challenged with control RNA or *in vitro* transcribed EBER1 or EBER2. All experiments were performed at least 3 times and data were expressed as mean ± SD. *P < 0.05 between assigned groups.

Previous reports indicated that EBER1 could cause an IFN-β inducible immune response through TLR3 signaling in infectious mononucleosis, chronic active EBV infection and EBV-associated hemophagocytic lymphohistiocytosis [9]. We therefore investigated the signaling pathways induced by EBERs in NPC cells. TLR3 expression was significantly stimulated by *in vitro* transcribed EBER1 or EBER2 in HNE2 cells (Figure 2A) and EBER1 or EBER2 stimulation of TLR3 transcription was in a dose-dependent manner (Figure 2B). To verify whether TLR3 is required for EBER induced inflammatory response, shRNA constructs targeting TLR3 was devised and verified (Figure 2C). Lenti-sh-TLR3#1 transduced HNE2 cells were used to examine the effect of down-regulation of TLR3 on cellular response to EBERs by qPCR and ELISA. Intriguingly, EBER1 or EBER2 triggered inflammation was overwhelmingly relieved in the TLR3 knockdown cells, which gave rise to much less TNFα and IL-6 transcripts and release into cell culture supernatant compared with the control cells (Figure 2D–2E). Consistent with the observation in HNE2 cells (EBV negative), knockdown of TLR3 in C666–1 cells (EBV positive) showed a similar reduction of TNFα expression (Figure 2F). To further characterize the involvement of TLR3 in the activation of EBERs triggered inflammation, we utilized a novel TLR3/dsRNA complex inhibitor [18] to disrupt EBER-TLR3 interaction in C666–1 cells. Treatment with the inhibitor led to a decrease in TNFα transcripts and release into the culture supernatant (Figure 2G).

NFκB pathway has previously been shown to be activated in response to TLR3 signaling [19]. We therefore investigated whether EBERs could activate NK-κB via TLR3 in NPC cells. Accordingly, an attenuation of TLR3 related IκBα signaling pathway was observed in TLR3 knockdown cells compared with the controls (Figure 2H). Thus, TLR3 is involved in and required for EBERs-induced inflammation in NPC cells.

### EBERs-LMP1-NFκB reciprocal signaling amlification loop enhances inflammation in NPC cells

**Figure 3:**
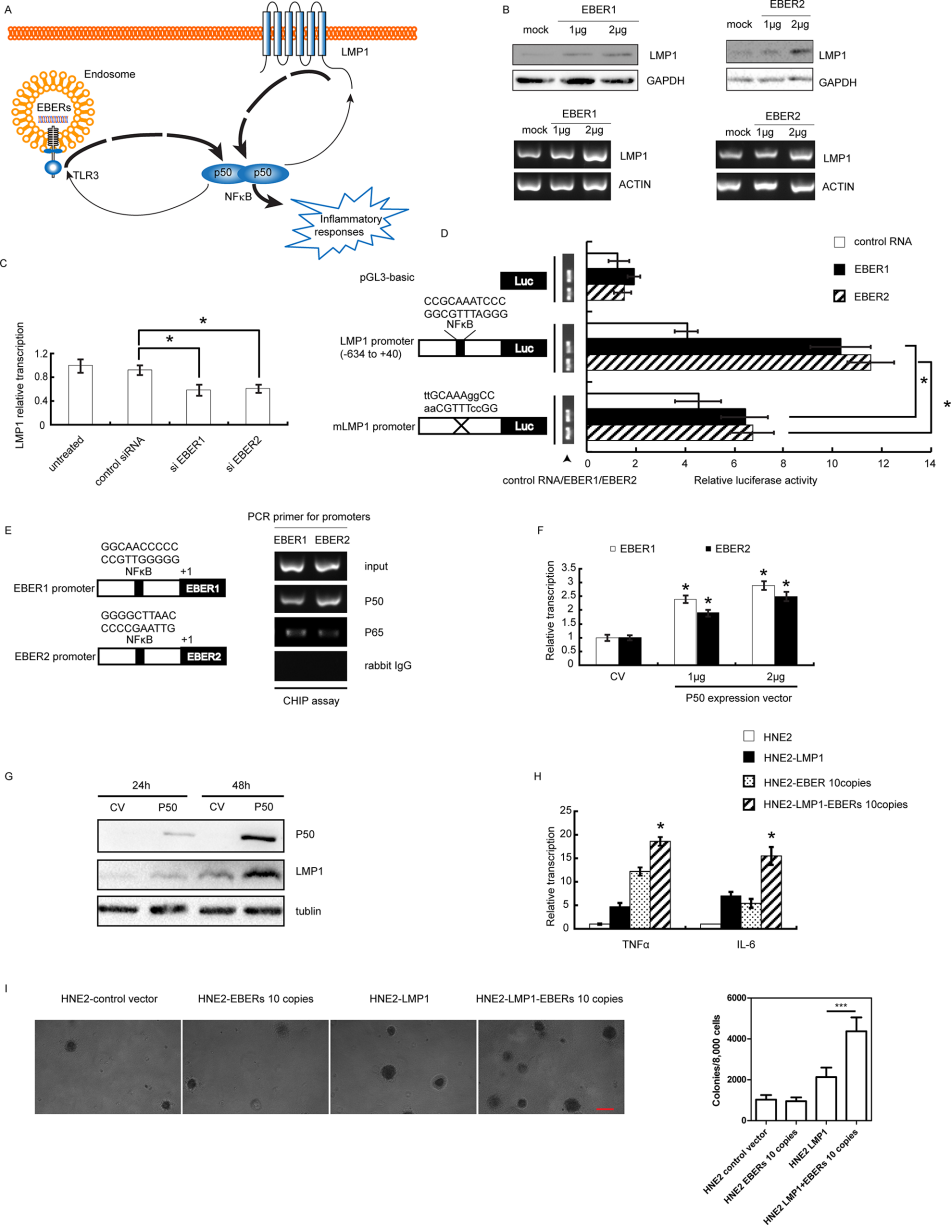
EBERs-LMP1 interactive positive feedforward loop amplifies the inflammatory response in NPC cell lines **A.** Schematic description of a proposed model for EBERs-LMP1 interactive feedfroward loop in promoting cancer-related inflammation. EBERs and LMP1 can both trigger moderate inflammatory response through NFκB as a key node. In addition to the role as an inflammation mediator, NFκB can transcriptionally up-regulate EBERs and LMP1. As a result, EBERs promote LMP1 transcription via NFκB which in turn transcriptionally induce more EBERs expression via NFκB likewise. Consequently, EBERs and LMP1 synergistically generate overwhelming signals to promote NFκB mediated cancer-related inflammation. **B.** C666–1 cells were challenged with *in vitro* transcribed EBER1 or EBER2 for 24 h and LMP1 expression was determined by western blotting and RT-PCR. **C.** LMP1 transcription level was assayed by qPCR in C666–1 cells treated with EBER1 [14]or EBER2 siRNAs for 48 h [14]. **D.** *In vitro* transcribed EBER1 or EBER2 was co-transfected with Renilla reniformis expression plasmids driven by LMP1 promoter with or without mutation in NFκB binding site and pGL3-basic was used as a control. 24 h later, luciferase activity was measured. Wild-type or mutated sequences of NFκB binding site in LMP1 promoter are indicated and the mutated bases are in lowercase. **E.** ChIP assay was performed to measure occupancy of EBER promoter by NFκB using antibodies against p50 and p65 in C666–1 cells. Putative NFκB binding sites of EBER1 and EBER2 are shown in the left panel. **F.** C666–1 cells were transiently transfected with p50 expression plasmid for 48 h, and EBER1 or EBER2 transcripts was determined by qPCR. **G.** LMP1 expression was analyzed by western blotting in C666–1 at 24 and 48 h post transfection with p50 expression plasmid. **H.** mRNA levels of TNFα and IL-6 were determined in HNE2 and HNE2 cells stably expressing EBERs or/and LMP1. **I.** Soft agar assay was conducted to demonstrate the anchor-independent growth ability conferred by LMP1 or/and EBERs. Experiments were conducted three times and the data were expressed as mean ± SD. *P < 0.05 versus mock treated group in (F) or between assigned groups as indicated in (C), (D) and (I) In (H) *P < 0.05 between HNE2-LMP1-EBERs cells and any other groups for TNFα and IL-6. CV, control vector.

LMP1, a well-recognized initiator of oncogenic pathway in EBV-infected NPC, has been shown to activate the NFκB, JNKs, JAK3, MAPKs signaling pathway to promote NPC cell proliferation, radioresistance and immune resistance [20–22]. Previous studies also suggested that NFκB could bind to the LMP1 promoter to enhance the oncogenic potential of LMP1 [23]. Interestingly, through bioinformatics analysis we found the existence of an NFκB binding site in both EBER1 and EBER2 promoters (Supplementary Figure S5). In consideration of these observations, we propose that EBERs may cooperate with LMP1 through the NFκB mediated signaling amplification loop to augment the inflammatory response in NPC (Figure 3A). To establish this reciprocal regulatory mechanism, we first examined the effect of EBERs on LMP1 expression at both RNA and protein levels. As shown in Figure 3B, *in vitro* transcribed EBER1 or EBER2 could increase the level of LMP1 expression in a dose dependent manner. Treatment of C666–1 cells with siRNA for EBER1 or EBER2 decreased LMP1 transcription level (Figure 3C). To elucidate EBER-mediated up-regulation of LMP1 via NFκB, we constructed the pGL3 plasmid composed of luciferase reporter gene driven by LMP1 promoter (−634 to +40) encompassing the NFκB binding site with or without mutation. When HNE2 cells were co-transfected with *in vitro* transcribed EBER1 or EBER2 and the reporter gene constructs, the luciferase activity driven by the wild type LMP1 promoter increased more than two fold relative to unchallenged cells, while this increase was greatly diminished when the NFκB binding site was mutated (Figure 3D). We then further verified the direct binding of NF-κB to the EBER promoters by a CHIP assay, showing that p50 rather than p65 was the main subunit participated in EBER1 or EBER2 transcription regulation (Figure 3E). Transfection of EBV positive C666–1 cells with p50 expression vector led to an approximately 2 fold increase of EBER1 and EBER2 transcripts (Figure 3F) and significant increase in LMP1 expression (Figure 3G) compared with the vector transfected cells.

To test whether the EBER-NFκB-LMP1 reciprocal signaling amplification loops exist in NPC cells, the cooperative stimulation of inflammatory cytokine production by EBERs and LMP1 was measured in LMP1 expressing HNE2 cells that were challenged with or without *in vitro* transcribed EBER1 or EBER2. Indeed, levels of TNFα and IL-6 in LMP1-EBER expressing HNE2 cells were among the highest compared with that in HNE2, HNE2-LMP1 and HNE2-EBER cells (Figure 3H). In further support of our observations, soft agar assay showed that, while EBERs alone appeared not to increase anchor-independent growth ability of HNE2, the cooperative action of EBERs and LMP1 could obviously increase this ability (Figure 3I). Taken together, our results suggest that in EBV infected epithelial cells EBERs and LMP1 cooperate to amplify regulatory loop with NFκB as a key node to enhance the inflammatory response.

### EBERs-induced inflammation promotes tumor growth

**Figure 4:**
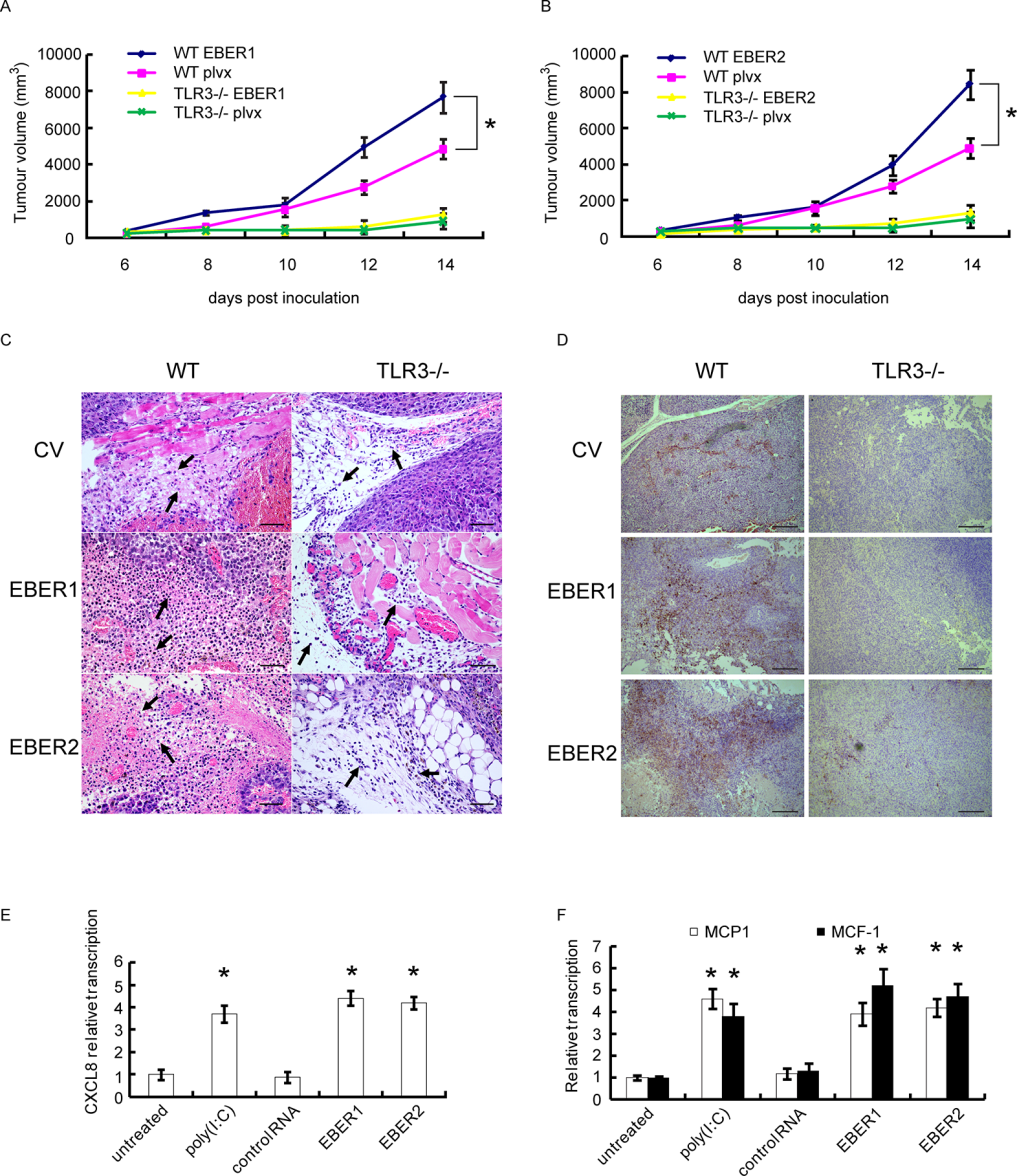
Effect of EBERs-TLR3 on B16 tumor growth and macrophage infiltration **A** and **B.** B16 cells stably transduced with lentiviral vectors expressing EBER1 (A) or EBER2 (B) were inoculated into WT or TLR3^−/−^ C57 BL/6 mice. B16 tumor growth curves were presented (*n* = 5). plvx is the empty lentiviral vector control. **C.** Monocyte infiltration (where the arrows pointed) represented by HE staining of B16 tumor slides derived from (A) and (B) Scale bar: 25 μm. **D.** Macrophage infiltration represented by IHC assay where the tissues were stained with CD68 antibody as shown in brown. Scale bar: 100 μm. **E.** HNE2 cells were stimulated with EBER1, EBER2, control RNA and Poly(IC) for 24 h, from which the culture supernatant were collected and added to U937 cells for 24 h. RNA from U937 cells was extracted and expression of CXCL8 was determined by qPCR. **F.** MCP-1 and M-CSF status were analyzed by qPCR after HNE2 cells were transfected with *in vitro* transcribed EBER1 or EBER2. All experiments were performed at least three times and representative data are presented. In (A) and (B), *P < 0.05 between assigned groups. In (E) and (F), *P < 0.05 between untreated/control and any other groups.

To determine *in vivo* whether EBERs could promote tumor growth through TLR3 signaling pathways, we used TLR3 knockout mice (TLR3^−/−^) to verify the impact of EBERs on growth of syngeneic tumor (B16, mouse melanoma cells). EBER1 or EBER2 stable expressing B16 cells were inoculated s.c. into wild-type (WT) or TLR3 gene knock-out C57 mice and tumor growth were recorded. As shown in Figure 4A and 4B, B16 tumors expressing EBER1 or EBER2 grew faster than the vector transduced B16 controls in WT mice (*P* < 0.05), while this discrimination was not observed in TLR3^−/−^ groups. Notably, all the B16 tumors in WT group grew about 2 times faster than that in TLR3^−/−^ mice, which indicates TLR3 is favorable for tumor growth *in vivo*.

Solid tumor development is facilitated by its microenvironment, which is composed of tumor associated macrophages (TAMs), DCs and Th2-deviated CD4+ T cells that release tumor-promoting cytokines [24]. To examine *in vivo* whether EBERs trigger inflammatory response, we analyzed the tumor tissues from WT and TLR3^−/−^ mice. HE staining indicated that there were more monocytes infiltrated in the tumor parenchyma of the EBER expressing tumors compared with the controls in WT group, while in TLR3^−/−^ mice, much less infiltrated monocytes were seen in tumors (Figure 4C). Furthermore, immunohistochemical analysis of CD68, a marker for mouse macrophages, confirmed that more macrophages infiltrated in EBERs-expressing tumors in WT mice in comparison with the tumors in TLR3^−/−^ mice or in WT mice of the vector controls (Figure 4D).

Production of CCL17, CCL1, chemokine (C-X-C motif) ligand-13 (CXCL13) and CXCL8 (IL-8) has been shown to be associated with the activation program for M2 tumor associated macrophage (TAM), and is parts of mononuclear phagocyte-mediated regulatory circuits of innate and adaptive immunity [25]. To determine the molecules responsible for macrophage activation, we collected the supernatants from HNE2 cells that were challenged with *in vitro* transcribed EBER1 or EBER2. Incubation of the supernatants with U937 cells (a macrophage cell line) resulted in up-regulation of CXCL8 in U937 (Figure 4E). In addition, we also found that EBERs could activate MCP-1 and M-CSF transcription in HNE2 cells (Figure 4F), which has been suggested to be responsible for TAM polarization [26].

**Figure 5:**
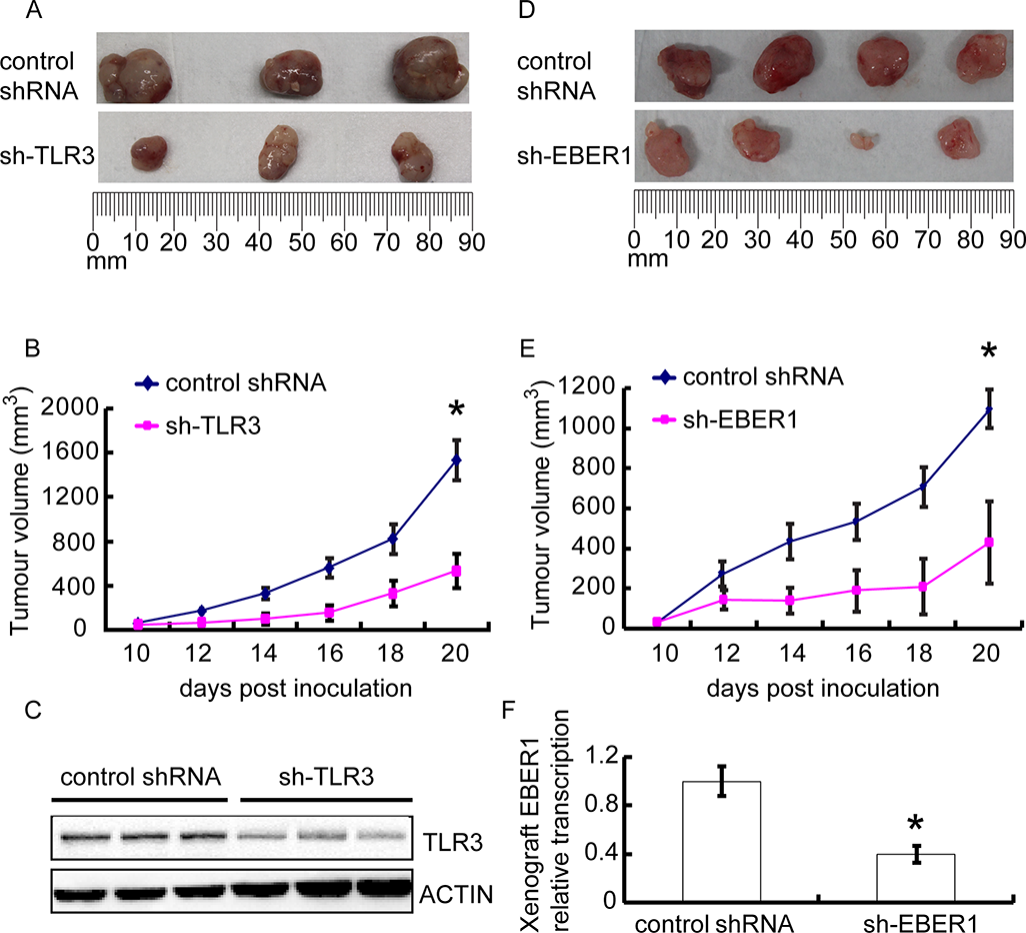
Effect of EBER-induced inflammation via TLR3 on human NPC xenograft growth *in vivo* C666–1 cells transduced with TLR3 shRNA **A–C.** or EBER1 shRNA **D–F.** were injected subcutaneously into female nude mice, and the tumors were allowed for growth for 20 days. (A) and (D) images of xenograft tumor size at day 20. (B) and (E) the xenograft growth curves. *N* = 3 or 5 respectively. (C) and (F) indicated that TLR3 or EBER1 were efficiently down-regulated in xenografts. Data are representative of three similar experiments. *P < 0.05 versus control group in (B), (E) and (F)

To further evaluate *in vivo* whether EBER-induced inflammation via TLR3 promotes tumor development, we subjected EBV-positive NPC C666–1 cells to tumor formation assay in nude mice. When TLR3 was knocked down with shRNA in C666–1, tumor growth was markedly inhibited (*P* < 0.05) (Figure 5A-5B). Western blots confirmed the silence of TLR3 expression in tumor tissues (Figure 5C). Similarly, when EBER1 was down-regulated in C666–1 with shRNA, tumor formation was also decreased relative to the controls (*P* < 0.05) (Figure 5D–5E). The reduced level of EBER1 in sh-EBER1 xenografts was demonstrated by qPCR (Figure 5F).

In sum, the results presented herein demonstrate that through the recognition by TLR3, EBERs can induce tumor cells to produce cytokines *in vivo* that potentially recruit and activate macrophages, leading to a protumorigenic microenvironment for solid tumor growth.

### EBERs are involved in TNFα induction in NPC patients and correlate with poor prognosis

**Figure 6:**
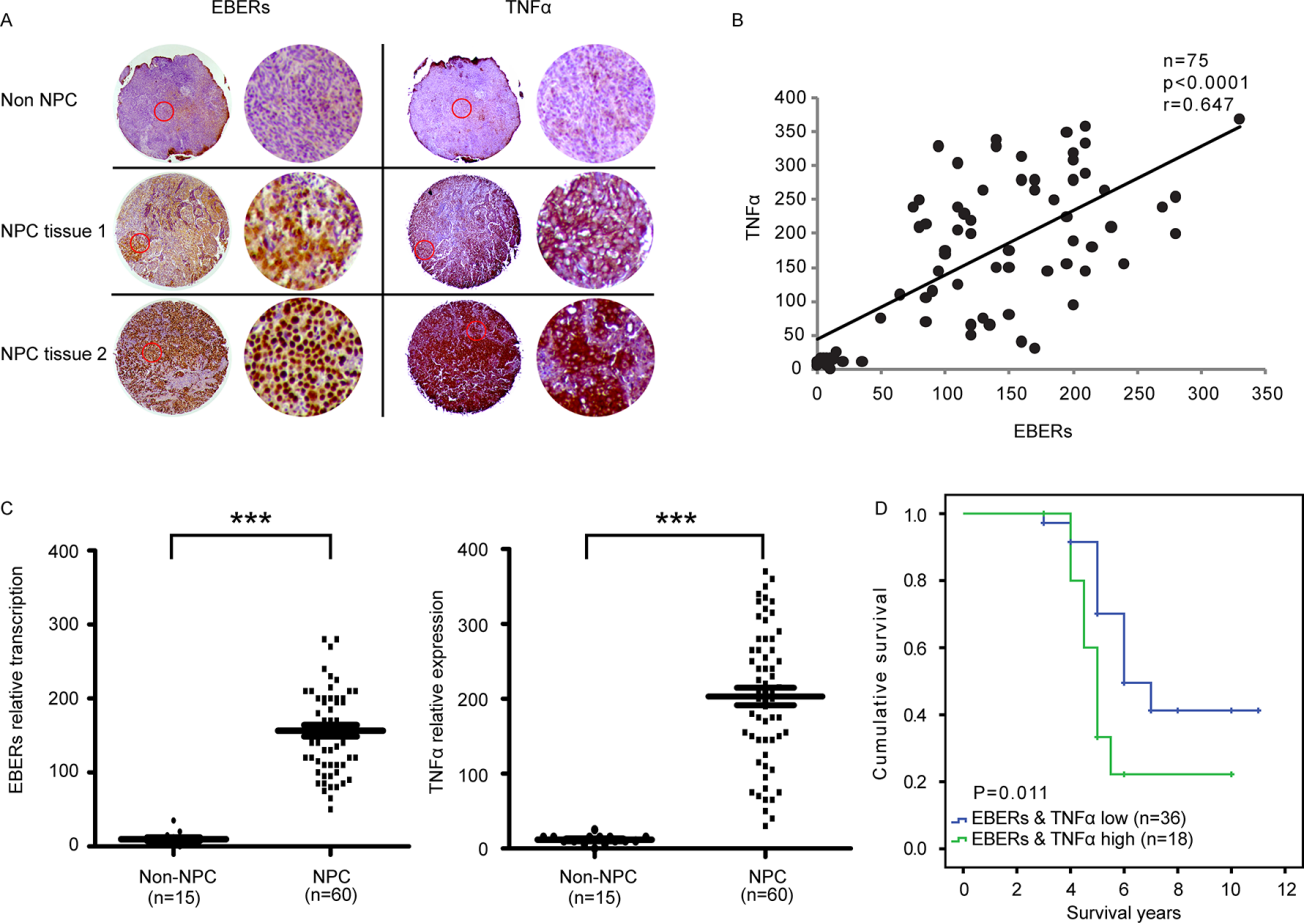
Correlation of EBERs with TNFα in NPC patients and its clinical relevance **A.** Representative images of three sections from two cancerous and one non-cancerous tissues hybridized with EBERs probe (Left panel) or stained with anti-TNFα antibodies (Right panel). Each panel are composed of two columns: The left displays the original images of specific tissues and the right represents the enlarged version of typical area enclosed in red circle of corresponding tissue in the left column. **B.** Correlation analyses of EBERs and TNFα based on ISH and IHC scores from the NPC tissue array (Cancerous tissues NO.: 60; non-cancerous tissues NO.: 15). **C.** Analysis of EBERs RNA and TNFα protein levels in non-cancerous and NPC tissues. **D.** Kaplan–Meier overall survival analysis based on the levels of EBERs and TNFα (“EBERs & TNFα low” cancerous tissues NO.: 36; “EBERs & TNFα high” cancerous tissues NO.: 18). The cutoff value for “EBERs & TNFα low” and “EBERs & TNFα high” is defined following the instructions at http://molpath.charite.de/cutoff [45]. ***P < 0.001 between non-NPC group and NPC group.

To corroborate our findings in clinical samples, we examined the association of TNFα and EBER expression in NPC tumor tissues. By analyzing a tissue array composed of 15 non-tumor tissues and 60 NPC tissues with the detailed clinical records (Pantomics), we found a positive correlation of levels between EBERs and TNFα (*P* < 0.0001, *r* = 0.647) (Figure 6B). Representative images of non-NPC and NPC tissues were displayed in Figure 6A, which were either immunohistochemically stained for TNFα or *in situ* hybridized with an EBER probe. We next asked whether the transcription level of EBERs and expression of TNFα were clinically correlated. As shown in Figure 6C, EBERs transcription level and TNFα expression levels were elevated in tumors compared with non-tumor specimens (*P* < 0.0001). Furthermore, the Kaplan–Meier overall survival curves showed that the combination of EBERs and TNFα expression was a potential predictor of the poor survival of NPC patients (*P* = 0.011) (Figure 6D). Thus, these data suggest that in EBV positive NPC, high level of EBERs induces inflammatory cytokine production, such as TNFα, which in turn promotes NPC development.

## DISCUSSION

A growing number of chronic inflammatory conditions have been described where the initial trigger is not well defined but does seem to involve infection. EBERs have been investigated in lymphoma with their ability to impact on tumor biology such as anti-apoptosis and protein translation [5, 6]. In the case of NPC, a few reports indicate that EBERs could resist apoptosis triggered by Poly(I:C), promote growth ability via IGF and affect lipid metabolism through low-density lipoprotein receptor (LDLR) and fatty acid synthase (FASN) [27–29]. However, controversial conclusions were made with EBERs’ participation in the process of transformation [27, 30]. In truth, reports are also controversial regarding to EBER's capacity to transform lymphocyte [31]. Here, we show that EBERs are strong inducers for inflammatory response in NPC, suggesting that EBERs may be responsible, at least in part, for chronic nature of the EBV-induced inflammation in nasopharynx that leads to cancerous lesions.

Most solid malignancies trigger an intrinsic inflammatory response that build up a pro-tumorigenic microenvironment [32]. Our work provides new insights in EBER-associated inflammation through TLR3 pathways. We showed that EBER expression in NPC resulted in stimulation of inflammatory cytokine release that caused infiltration of TAMs in NPC xenograft and TLR3 KO models. Our data establish a crucial role for both EBER1 and EBER2 in sustaining inflammatory signaling through TLR3 and in enriching tumor microenvironment. A previous study reported that EBER2 was not found to be secreted in the context of lymphomas [33], which is in discrepancy from our observation in which both EBER1 and EBER2 were present in the form of exosomes. This may reflect the pathological differences between carcinomas of epithelial origin and lymphomas.

The transcription regulation of EBERs is fascinating in consideration of their being the most abundant transcripts. c-Myc, ATF-2 and TFIIIC have been reported to affect EBERs’ basic transcription process [34, 35] and EBNA1 is involved in EBERs transcription regulation through induction of cellular transcription factors [36]. Given the significance of NFκB in cancer-related inflammation, we explored its participation in EBER's transcription and verified that p50 was the major subunit of NFκB that could bind EBER1 or EBER2 promoter. To our knowledge, this is the first report with regard to the EBERs’ regulation by NFκB.

It has been suggested that the cross-talking between TLRs and other signals is an important feature of the pathogen-host interaction as it is likely to occur *in vivo* during infection and inflammation [37]. Signaling pathways that mediate the pro-tumorigenic effects of inflammation are often subject to a feed-forward loop [38]. In the present study, we provide evidence that EBERs activate the TLR3 pathway, which results in inflammatory cytokine production via NFκB, while in the same EBV infected cells, the viral oncoprotein LMP1 also stimulates NFκB and generates proliferative signals. Thus, we postulate that the positive regulatory loop between EBERs and LMP1 via NFκB may form a necessary driving force for inflammation-to-carcinogenesis transition in EBV infected epithelial cells. Consistent with this notion, tumor-related signal is involved in the development of chronic inflammation in animal models and patients [39]. Of note, LMP1 was reported to be the exclusive oncoprotein encoded by EBV, however, its expression in immortalized NP cells is not tumorigenic when injected subcutaneously into athymic nude mice [40], which infers other additional genetic events are required to complete the process of malignant transformation of NP cells. In light of the observation that EBERs alone probably may not be able to transform NP cells [27], thus it's intriguing to explore the function of EBERs-LMP1 signaling amplification loop in the process of malignance. Furthermore, recent observations suggest inflammation regulatory circuit may actually initiate cellular transformation [41, 42]. Accordingly, it's reasonable to conceive the potential transformation ability of EBERs-LMP1 regulatory circuit.

Another significant question relating to the function of EBERs is whether they are instrumental for or even capable of cell transformation. A previous paper denied this possibility [31] and in support of this, a more recent report demonstrates that neither EBER is essential for transformation efficiency or growth of lymphoblastoid cell lines (LCL) [33]. In accordance with these studies, soft agar assay conducted in our present study indicated EBERs could not confer anchor-independent growth ability to HNE2 cells, an indirect indicator of epithelial cell transformation capacity. Thus it's inferable that EBERs, despite their abundance and reported interaction with several important proteins, may execute an auxiliary role in cell malignancy process via non-resolving inflammation. This auxiliary effect could become predominant if additional genetic events co-exist, as shown in the present study where LMP1 is highly expressed.

Taken together, our results suggest that EBERs are instrumental for launching a vicious cancer-promoting inflammatory response represented by TNFα via TLR3 in vritro and *in vivo*. Moreover, we have made it clear that EBERs could amplify this inflammatory response via interacting with surrounding macrophages and modulate the transactivating feedforward loop with LMP1 rather than fulfill this response alone. Based on our observations, the oncogenic role of EBV is not simply due to any previously identified oncoprotein it encoded, instead the inflammation response triggered by EBER-TLR3-LMP1 regulatory loop should be the focus. Thus, interfering with this regulatory loop with TLR3 antagonist or targeted molecules for EBERs may provide a novel strategy for NPC prevention and treatment [43].

## MATERIALS AND METHODS

### Cells, transfection and stable transfectants

NPC cell lines HNE2 and C666–1, human monocytic cell line U937 and mouse melanoma cell line B16 were cultured in RPMI (Invitrogen, Carlsbad, CA, USA) supplemented with 10% fetal bovine serum, 100 units/ml penicillin and 100mg/ml streptomycin. Ten copies of EBER expression plasmid was constructed as previously described [13]. Stable transfectants in HNE2 cells were selected in 300 μg/mL G418. *In vitro* transcribed EBERs were produced using a T7 *in vitro* transcription kit (Promega, Fitchburg, WI, USA), followed the instructions provided by the manufacture. For transient transfection, cells were transfected with DNA plasmids or *in vitro* transcribed EBERs using LipoFectamine 2000 (Invitrogen, Carlsbad, CA, USA). for HNE2 cells treated with *in vitro* transcribed EBERs, given that the amount of introduced “exogenous EBERs” is critical for the relevance to the endogenous EBERs, we take into account of the molecular weight of EBER1 and EBER2 (approximate 56000–58000 dalton), the estimated copy number of EBER1 and EBER2 present in C666–1 (approximate 6 × 10^6^ or 6 × 10^5^ respectively) determined by absolute quantification with q-PCR (data not shown) and previous reports [44], the seeded cell number (5 × 10^5^ per well) and also the estimated transfection efficiency (approximate 40%-50%). Based on these parameters and the following equation “*in vitro* transcribed EBER amount applied to challenge HNE2 (g)=EBER copy number × EBER molecular weight × seeded cell number/Avogadro constant/transfection efficiency”, the amount of *in vitro* transcribed EBER that should be applied can be calculated. Thus 0.6 μg EBER1 or 0.1 μg EBER2 was applied to challenge HNE2 cells If the amount is not specifically mentioned. For RNAi experiments, siRNA targeting EBER1 or EBER2 was transfected twice for two consecutive days at 50nM using LipoFectamine 2000. Lentivirus production was carried out using the lenti-viral packaging kit (Clontech, Takara Bio Inc., Shiga, Japan). TLR3 stable knockdown cell line was generated by with lentiviral-based-shRNAs. The targeting sequences of siRNA/shRNA for EBER1, EBER2 and TLR3 were listed in Supplementary Table S1.

### ELISA

After stimulation, the expression of the inflammatory cytokines in culture supernatants was assessed using a commercially available Quantikine ELISA kit according to the manufacturer's instructions (R&D systems, Minneapolis, MN, USA).

### Immunoblotting

Cell and tissue lysates prepared in a standard RIPA buffer were separated in PAGE and proteins were transferred to NC membranes (Pierce Biotechnologies Inc., Rockford, IL, USA). Membranes were probed with primary antibodies for TLR3, NFκB family detection kit, IκB (Cell Signaling Technologies, Danvers, MA, USA), GAPDH, β-ACTIN (Santa Cruz, CA, USA) and secondary HRP-conjugated antibodies and developed using ECL detection reagent (Pierce, Biotechnologies Inc., Rockford, IL, USA).

### Quantitative RT-PCR

Total RNA was extracted from NPC cell lines and tumors. RNA samples were reverse transcribed using the SuperScript III First-Strand Synthesis SuperMix for qPCR Kit (Life Technologies, California, USA)., and quantitative real-time PCR (qRT-PCR) analysis was performed using the CFX-96 Real-time PCR System (Bio-Rad, Hercules, CA, USA). The primer sequences were listed in Supplementary Table S2.

### Transmission electron microscopy on exosomes

10 μl of exosome suspension in 1xPBS was dried onto freshly glow discharged 200 mesh formvar-carbon-coated copper grids, negatively stained with 2% aqueous uranyl acetate and observed with a Tecnai G2 Spirit TWIN transmission electron microscope (TEM) (FEI, Hillsboro, Oregon, USA).

### Luciferase assay

Reporter assay was conducted according to the manufacturer's directions (Promega, Fitchburg, WI, USA). The luciferase reporter plasmids were co-transfected with pRL-SV40 to correct for variations in transfection efficiency. The relative luciferase activity was normalized to the value of pRL-SV40 activity. The LMP1 promoter sequence incorporated with or without NFκB binding site mutation has been described previously [20].

### Chromatin immunoprecipitation (ChIP) assay

Cells were grown to 70–80% confluence in 10-cm plates, and were then processed for ChIP assays using a kit (EZ-ChIP, Millipore, Billerica, MA). DNA was sheared to a size of 200–1, 000 base pairs (bp) prior to performing the immunoprecipitation (IP). The sheared chromatin was pre-cleared by incubating with protein G beads for 1 h at 4°C to reduce non-specific background. Pre-cleared chromatin was then incubated with anti-p65, anti-p50 (Cell Signaling Technologies, Danvers, MA, USA) or anti-rabbit IgG (2 mg, Santa Cruz, CA, USA), or no antibody overnight at 4°C, respectively. Protein G bead incubation, washing of ChIP reactions, DNA elution from protein G, cross-link reversal, RNA removal and purification of eluted DNA were performed following the protocol included in the kit. Isolated DNA was subjected to PCR to detect the binding of NFκB subunits with EBER1 promoter. The primers for the EBER1 promoter domain, which included the NFκB binding region, that were used in the ChIP assays were shown in Supplementary Table S1.

### Anchorage-independent growth assay

(8 × 10^3^/mL/well) were seeded into 6-well plates with 0.3% Basal Medium Eagle agar containing 10% FBS and cultured. After 2 weeks of incubation at 37° C with 5% CO_2_, the number of colonies formed was determined.

### Mouse models

All the experimentation for animals was approved by Animal Ethics Committee of Central South University, following the Guidelines of Animal Handling and Care in Medical Research from Hunan Province, China. For human NPC xenograft model, 6–8 weeks old female nude mice were from Vital River Laboratories, and the original breeding pairs were purchased from Charles River. 5 × 10^6^ C666–1 cells stably transduced with or without EBER1/TLR3 shRNA lentivirus were inoculated into the left flank of the mice for NPC tumor formation. For B16 synergetic tumor model, pathogen-free female C57BL/6 mice (6–8 weeks old, wild-type or TLR3 knockout TLR3−/−, from Jackson Lab) were injected subcutaneously with 200 μl of the cell suspension containing 3 × 10^5^ cells at the left flank of the mice.

### Immunohistochemistry assay

The samples of NPC tissue arrays or syngeneic tumor were subjected to heat-mediated antigen retrieval in 0.01 mol/l citrate buffer (pH 6.0). After cooling to room temperature, samples were treated successively with 1% methanol/30% H_2_O_2_ to block endogenous peroxidase and 5% bovine serum albumin to block nonspecific binding sites. After rinsing with PBS, they were incubated overnight at 4°C with the goat-anti-TNFα or rat-anti-CD68 primary antibody (Abcam, Cambridge, UK). The sections were then rinsed with PBS and incubated with the HRP conjugated secondary antibody (Abcam, Cambridge, UK) for 30 min at room temperature. Then the sections were washed and incubated with DAB (Abcam, Cambridge, UK), and was terminated by rinsing with distilled H_2_O. Finally, the samples were counterstained with hematoxylin.

### *In situ* hybridization

ISH was performed using the EBERs HRP-conjugated probe and DAB as substrate from ISH kit (Life Technologies, California, USA), according to the manufacturer's instructions.

### Statistical analyses

Statistical analyses were performed using SPSS 18.0 (SPSS) and GraphPad Prism, version 5. Results are expressed as means ± SD. For comparisons between groups, statistical analyses were performed using Mann-Whitney test. Differences with *P* values of <0.05 were considered significant. The significance of Kaplan–Meier survival analyses was determined using log-rank tests. Spearman's rank correlation coefficient was used to evaluate the correlation between IHC analyses of TNFα and ISH results of EBERs (staining intensity × percentage). All statistical tests were 2-sided.

## SUPPLEMENTARY FIGURES AND TABLES


