# Supplementary material for: EBV-encoded RNA via TLR3 induces inflammation in nasopharyngeal carcinoma
Source: Oncotarget. 2015 Jul 4;6(27):24291–303. doi: 10.18632/oncotarget.4552 (PMC4695186; doi:10.18632/oncotarget.4552)
Supplement: Supplementary file 1 [file oncotarget-06-24291-s001.pdf]

SUPPLEMENTARY FIGURES AND TABLES

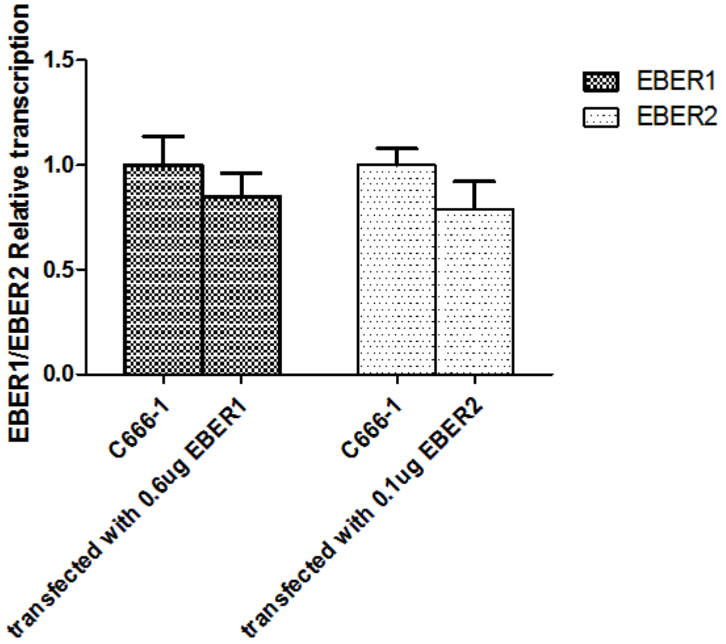

Supplementary Figure S1: Validation of comparable EBER1 and EBER2 levels between the HNE2 cells challenged with *in vitro* transcribed EBER1 or EBER2 and C666-1 cells.

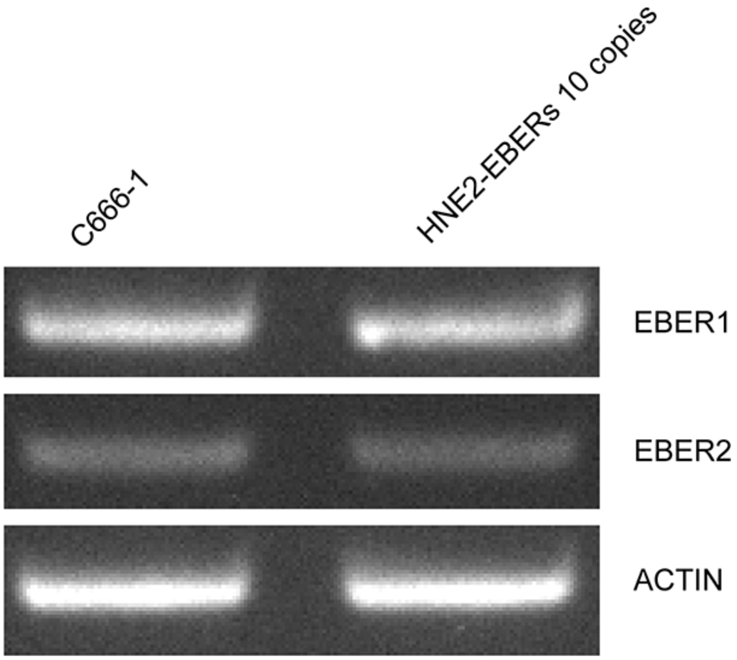

Supplementary Figure S2: Identification of EBER1 and EBER2 transcription level in HNE2 cells expressing 10 copies of EBERs construct relative to C666-1 cells.

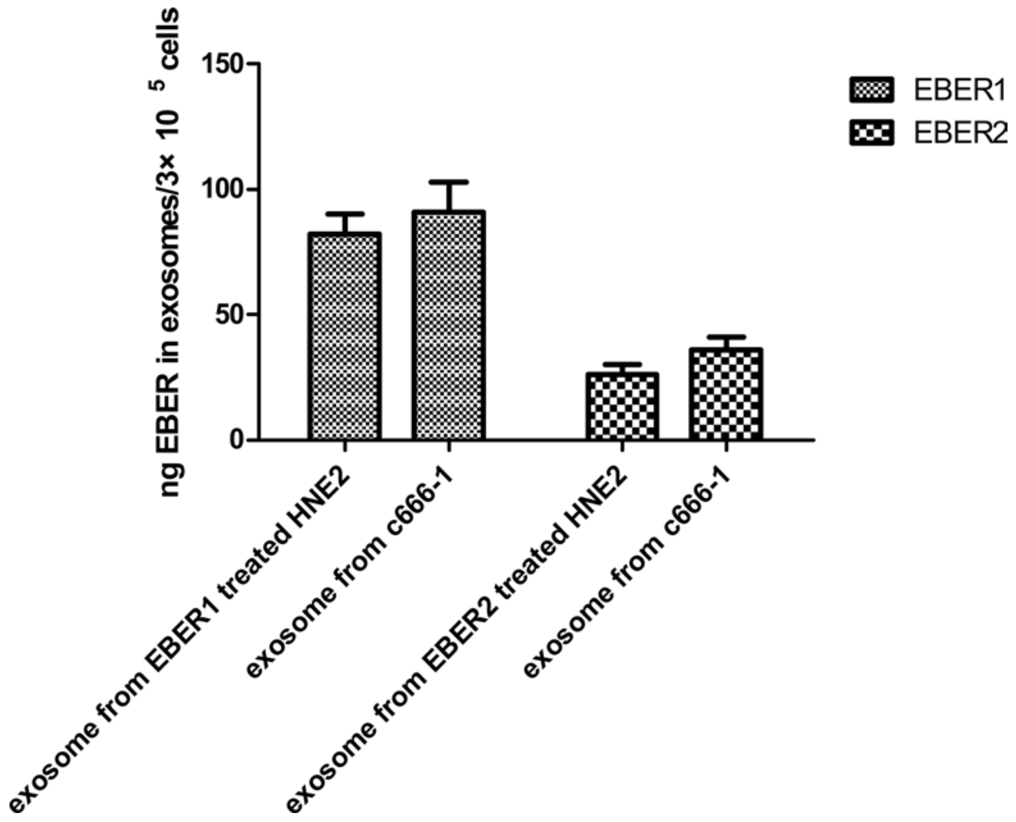

Supplementary Figure S3: Comparison of the amount of EBER1 and EBER2 in isolated exosomes from C666-1 and *in vitro* transcribed EBERs treated HNE2 cells.

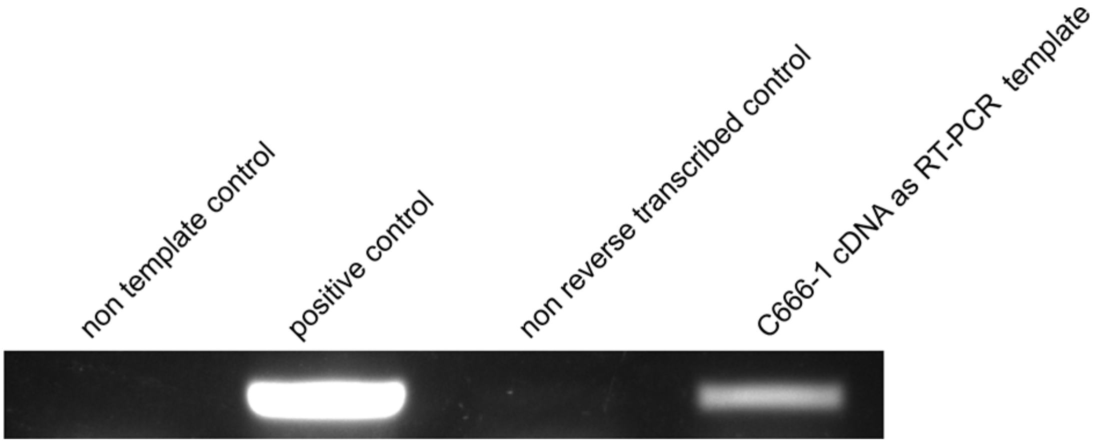

Supplementary Figure S4: EBER1 and EBER2 may coexist as a transcript. Possible existence of a transcript encompassing both EBER1 and EBER2 was identified by RT-PCR with EBER1 forward primer and EBER2 reverse primer. Positive control: EBERs expression plasmid as PCR template; Non reverse transcribed control: C666-1 RNA treated with DNase I as PCR template.

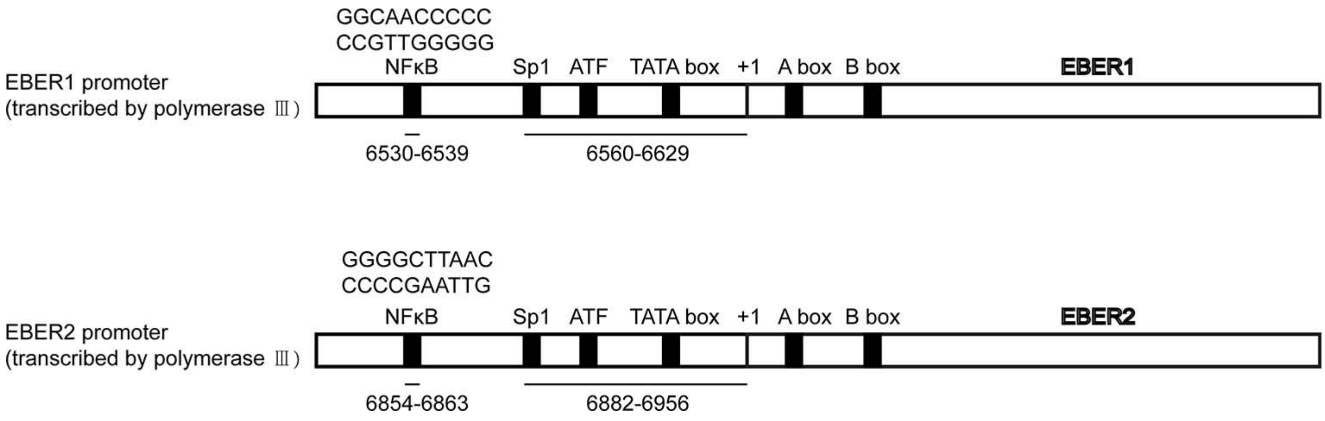

Supplementary Figure S5: Graphic representation displays EBERs transcripts and location of EBERs transcription factor.

Supplementary Table S1. Primer sequences

Gene	Forward	Reverse
EBER1	5′-AGGACCTACGCTGCCCTAGA-3′	5′-AAAACATGCGGACCACCAGC-3′
EBER2	5′-AGGACAGCCGTTGCCCTAGTG-3′	5′-AAAATAGCGGACAAGCCGAATAC-3′
EBER1 (CHIP)	5′- TGTAAAACACCGACCGC -3′	5’- AAGACAGACTACGTCACCGT-3’
EBER2 (CHIP)	5′-TATCCCGCAGTTCCAC-3′	5′-TGATGTAGCGAACCGTAA-3′
M-CSF	5′-TAGCCACATGATTGGGAGTG-3′	5′-TATCTCTGAAGCGCATGGTG-3′
MCP-1	5′-GCTGTGATCTTCAAGACCATTGTG-3′	5′-TGGAATCCTGAACCCACTTCTG-3′
IL-6	5′-GGTACATCCTCGACGGCATCT-3′	5′-GTGCCTCTTTGCTGCTTTCAC-3′
TNFα	5′-CCCAGGCAGTCAGATCATCTTC-3′	5′-AGCTGCCCCTCAGCTTGA-3′
IL1 α	5′-CGCCAATGACTCAGAGGAAGA.-3′	5′- AGGGCGTCATTCAGGATGAA-3′
TLR3	5′-CCTGGTTTGTTAATTGGATTAACGA-3′	5′-TGAGGTGGAGTGTTGCAAAGG-3′
IFNβ	5′-CAGCAATTTTCAGTGTCAGAAGC-3′	5′-TCATCCTGTCCTTGAGGCAGT-3′
LMP1	5′-AGCGACTCTGCTGGAAATGAT-3′	5′-TGATTAGCTAAGGCATTCCCA-3′
CCL1	5′-ACAGAAAGGCCCTCAACTGTC-3′	5′-TCCTTGATGTACTCTTGGACCC-3′
CCL17	5′-AGAGCCACAGTGAGGGAGAT-3′	5′-TTAATCTGGGCCCTTTGTGC-3′
CXCL13	5′-GCTTGAGGTGTAGATGTGTCC-3′	5′-CCCACGGGGCAAGATTTGAA-3′
CXCL8	5′-GAATGGGTTTGCTAGAATGTGATA-3′	5′-CAGACTAGGGTTGCCAGATTTAAC-3′
ACTIN	5′-CTGGAACGGTGAAGGTGACA-3′	5′-AAGGGACTTCCTGTAACAATGCA-3′

Supplementary Table S2. Targeting sequences for shRNA

Gene	Targeting sequences
EBER1	#1: 5′-CGGTGTCTGTGGTTGTCTT-3′; #2: 5′-CTCTGCTTTCTGCCGTCTT-3′
EBER2	#1:5′-GCATTTGCAAGTCAGGATT-3′; #2: 5′-GGATTCTCTAATCCCTCTG-3′
TLR3	1#: 5′-AAGTGCACTGTTAGCCATGAA-3′, 2#: 5′-CCTTACACATACTCAACCTAA-3′;3#: 5′-CCTCTTCGTAACTTGACCATT-3′,
